# Saving the debate: why psychological accounts of personhood ought not accept a univocal biological definition and criterion of death

**DOI:** 10.1007/s11017-025-09718-1

**Published:** 2025-06-10

**Authors:** Christopher Buford

**Affiliations:** The University of Akron, Akron, OH USA

## To the Editor

In 2019, David Hershenov argued for a set of three connected claims in this journal [1]. First, any psychological account of what it is to be a human person (i.e. a view denying that human persons are identical to human animals), need not and in fact *should not* formulate a definition of death distinct from the merely biological definitions of death typically offered for human animals [2–4]. Second, a distinct criterion for the deaths of human persons and human animals is also unnecessary as a biological criterion is capable of playing the role for both. Finally, accepting distinct conditions for ceasing to exist is sufficient to ground distinct accounts of the deaths of human persons and human animals. If Hershenov is correct, what appear to be important and significant disagreements in bieothics (e.g., whole brain theorists vs. higher brain theorists; animalists vs. psychological essentialists) are otiose. Hershenov’s arguments are thus worth examining. I argue in what follows that only the third claim withstands critical scrutiny.

It may help to start with the third claim to begin to understand Hershenov’s position. This claim is clearly the least controversial of the set. The difference in conditions for ceasing to exist between person and animal is required by the psychological theorist’s belief that human persons have certain mental capacities essentially, while human animals do not. If this is right, then a human person might go out of existence, but the human animal that previously overlapped with that person would remain. And this possibility entails a difference in the conditions sufficient for ceasing to exist. In such a case, the going out of existence of the human person occurs before the going out of existence of the human animal.

Consider now the first claim that only a biological definition of death is required, even if one is a psychological theorist. While the correct definition of death is of course not a settled matter, assume that a proponent of the psychological account accepts Bernat’s definition of the death of the human animal as “the permanent cessation of functioning of the organism as a whole” [4, p. 17]. As noted above, Hershenov argues that the defender of the psychological approach should also accept this as the correct definition of the death of a human person, even though the person on this view is distinct from their overlapping animal.

Given the psychological theorist’s commitment to a difference in the sufficient conditions for ceasing to exist discussed above, one may wonder how the same definition of death can apply to both the person and their animal. Hershenov accepts that when higher-order capacities required for the continued existence of the person cease, then the human person will also cease to exist. Yet he argues that a biological definition of death can fully capture this death as the person has, in virtue of ceasing to exist, also ceased to instantiate the biological processes associated with life. Further, since the human person has ceased to instantiate the biological processes associated with life, the person has died. Thus, according to Hershenov, there is no need for the proponent of the psychological account to insist on a definition of death distinct from the biological account that covers the death of their animal as well. Both the human person and human animal can die in the same way, by satisfying the same exact definition of death.

This is problematic for several reasons. First, Hershenov’s description of what transpires in the case of a cerebrum transplant itself appears to entail the need for multiple definitions of death. To see this, consider Hershenov on the transplant scenario.

Persons die when their bodies become inorganic or when they are reduced to the size of a cerebrum, because it is then that they cease to instantiate life processes. Imagine that all of the body is destroyed except the cerebrum, reducing the person from six feet and two hundred pounds to just a few inches and three or four pounds. Even if that organ is alive the person’s original life has ceased [1, p. 409].

Grant that the person’s (biological) life has ceased. The problem is that if there is only one univocal definition of death, then not only has this person’s life ceased, but the cessation must be *permanent*. But this need not be the case. Suppose that the person’s cerebrum is removed and the body is not destroyed. According to Hershenov, the person has died because they have ceased to instantiate life processes. What happens though if one returns the cerebrum to the animal? The person has not ceased to exist, so they now presumably once again instantiate life processes. Thus, they are alive. This means they were alive, then they died, and are now alive again. Biological death *for persons* thus need not be permanent.

Hershenov criticizes the psychological theorists attempt to identify at least two possible senses of ‘death.’ However, as was just shown, Hershenov’s favored position appears to countenance two distinct types of biological death. This is arguably even more problematic than some psychological theorists’ commitment to distinct definitions of death. It does not appear, for example, that other fundamental biological notions are treated in this way. Concepts such as *organism*, *life*, *organ*, etc. are treated univocally. Finally, I think there is some force to the objection that transplant scenarios, at least from the perspective of the psychological theorist, push back against the claim that ceasing to instantiate a life process is always a way of dying. Consider for example a successful cerebrum sway. On Hershenov’s understanding of what transpires, there are two deaths.

Hershenov’s second claim, that proponents of psychological accounts should, along with animalists, endorse a single criterion of death such as the whole-brain criterion of death, faces difficulties as well. It is important to note here that a criterion is to play a specific role in any comprehensive account of death. Bernat suggests that a successful criterion of death should have three key features. It should (i) provide necessary and sufficient conditions for death to occur (ii) be measurable and (iii) be capable of utilization in a death statute [4, p. 16]. Proposed criteria can thus be criticized on the grounds that they fail to meet (i), (ii), or (iii).

The claim that only a biological criterion of death is required faces a difficulty similar to that discussed concerning a definition of death. Namely, the whole-brain criterion is usually understood to require irreversible cessation of whole-brain function. However, if a person can survive (i.e., not cease to exist) a cerebrum transplant, then irreversible cessation of whole-brain function has not necessarily occurred for a successful transplant into a new human animal is a possibility. And thus, human persons and human animals are not governed by a single criterion of death. Thus, one will have a violation of condition (i). On Hershenov’s proposal, death will occur for human persons even though they fail to meet the condition of irreversible cessation of whole-brain function.

Notice as well that if one accepts that the same criterion of death governs both human persons and human animals, some deaths will not be measurable using the criterion itself. Thus, Bernat’s condition (ii) will not be satisfied. To see this, suppose that time *t* is when the human person dies and time *t* + when the human animal dies. Now imagine a case where, perhaps due to PVS, the human person dies prior to the death of the human animal. Thus, *t* is prior to *t*+. How is one to use the whole-brain criterion to identify *t* (i.e. the time of the death of the person)? The human animal’s brain is still functioning so one cannot rely on its lack of function.

Hershenov is aware of this consequence admitting on this account, “the whole-brain criterion and associated tests will not be diagnostically useful in determining the death of the *person* at bedside” [1, p. 413]. However, Hershenov does not explain how this is consistent with understanding the whole-brain criterion as a *genuine* criterion of death for human persons for it appears to fail to satisfy (ii) and (iii). An acceptable criterion of death does not just provide necessary and sufficient conditions; the notion of a criterion is partly epistemic. The correct criterion provides a way for medical professionals to reliably determine death to abide by normative principles such as the dead-donor rule. According to psychological accounts, the death of the person marks a significant event. And the whole-brain criterion is of little use in identifying when this occurs. Instead, it is the conditions associated with the higher-brain criterion that do. Thus, even if the person meeting the conditions stated in whole-brain criterion (*sans* irreversibility) are correlated with the death of the person, a higher-brain criterion will still be needed for measurement and presumably statutes if psychological accounts are correct. Thus, Hershenov’s claim that the whole-brain criterion can serve as the single criterion of death even if psychological accounts are correct is much too strong.

Hershenov argues that not only can psychological accounts accept a univocal biological definition and criterion of death, but that such accounts should as well. If my arguments are successful, psychological accounts should not, and in fact cannot, do so. Doing so would commit psychological theorists to accepting two distinct definitions of biological death. Further, a single biological criterion of death is incapable of fulfilling the role required of an appropriate criterion if psychological accounts are correct.
